# Facilitators’ experiences of co-designing an intrapartum care intervention in four sub-Saharan African countries: a qualitative study

**DOI:** 10.1136/bmjopen-2025-109931

**Published:** 2026-03-10

**Authors:** Erika A Saliba-Gustafsson, Nicole Sofia Rodriguez Neufeld, Claudia Hanson, Effie Chipeta, Helle Mölsted Alvesson

**Affiliations:** 1 Department of Global Public Health, Karolinska Institutet, Stockholm, Sweden; 2 Department of Disease Control, London School of Hygiene & Tropical Medicine, London, England, UK; 3 Department of Health Systems and Policy, Kamuzu University of Health Sciences, Blantyre, Southern Region, Malawi

**Keywords:** Africa South of the Sahara, Community-Based Participatory Research, Maternal medicine, Person-Centered Care, Quality Improvement, Implementation Science

## Abstract

**Abstract:**

**Objective:**

The aim is to explore co-design facilitators’ perspectives and experiences of using co-design to improve intrapartum care in four sub-Saharan African settings. The inquiry focuses particularly on how they fostered engagement, built trust and mitigated unintended consequences during the co-design process.

**Design:**

Qualitative interview study with reflexive thematic analysis.

**Setting:**

Sixteen public and private not-for-profit hospital-based maternity units in Benin, Malawi, Tanzania and Uganda (four per country).

**Participants:**

A total population sample of 10 co-design facilitators involved in a hospital-based co-design project implemented in maternity units in Benin, Malawi, Tanzania and Uganda were interviewed. Semistructured interviews were conducted between December 2022 and January 2023.

**Results:**

Co-design facilitators viewed co-design as a collaborative process to develop contextually relevant solutions. Our findings elucidate their role in facilitating consensus-building and fostering stakeholder ownership amidst significant power divides. They described approaches co-design facilitators take to maintain ongoing stakeholder engagement and manage misaligned expectations in a trusting and collaborative environment, while being mindful of existing tensions and power imbalances. They also highlighted key challenges faced, including navigating norms, power imbalances and unintended consequences.

**Conclusions:**

This study underscores the importance of power-sharing, fostering ownership and engaging end users equitably and continuously in co-design efforts, while also being aware of how to address its potential unintended consequences. Further research is needed to understand co-design facilitators’ impact on co-design and how to address unintended consequences for stakeholders during and after co-design activities in intrapartum interventions in low-resource settings.

**STRENGTHS AND LIMITATIONS OF THE STUDY**

- Facilitators were interviewed soon after completion of the main co-design phase, while they were still engaged in the project activities, minimising the risk of recall bias.
- Co-design facilitators interviewed received most co-design training online due to the COVID-19 pandemic, potentially influencing workshop delivery; however, their prior co-design experience—bolstered by an online platform for recorded sessions, cross-country discussions, resource sharing, and peer engagement during data collection and workshops—helped mitigate this.
- The interviewer was an outsider to the co-design facilitators interviewed and the project but an insider to participatory methodologies, which should have allowed interviewees to share their experiences more freely.
- Interviews were held online; while mostly effective they were conditioned on facilitators’ familiarity with digitally expressing uncertainty, methodological dilemmas and reflections.

## INTRODUCTION

 Co-design is increasingly being used in healthcare intervention development, actively involving patients and healthcare professionals as active partners to shape contextually relevant solutions.^1^,^9^ This approach empowers end users,^69^,^15^ leading to better implementation,^910 16^,^19^ improved workflows^1^ and sustained behaviour change.^20^ It also enhances co-design participants’ understanding of each other’s perspectives, provides a sense of community and promotes mutual accountability.^12^ Co-design can, however, be costly, time-consuming and tokenistic.^15 21^,^23^ It may also reveal existing tensions among individuals involved in the co-design process, which could lead to frustration and marginalisation.^5 22 24 25^ In fact, promoting meaningful engagement while managing conflicts, especially those stemming from power imbalances, is especially challenging in co-design.^22 26 27^ In healthcare, patients, for instance, may struggle to have their voices heard if healthcare professionals are involved,^27 28^ and patients may consequently be given a more passive decision-making role.^1 20^ Furthermore, despite co-design’s potential of transforming intervention development through inclusion and representation, involving a wide range of participants, particularly vulnerable or hard-to-reach groups, remains challenging.^5 11 15 29 30^ Their systematic exclusion risks further widening equity gaps.^30^ All participants need to feel their involvement is valued and important^5^ to support more representative inclusion.

Persons facilitating the co-design process, hereafter referred to as ‘co-design facilitators’, have a pivotal role in fostering trust, facilitating rapport-building, resolving conflicts and empowering participants to collaborate as equal partners.^1 8 9 22 25^ This helps minimise power struggles and supports intervention uptake by creating an environment that allows everyone equal opportunity to provide input and influence intervention design and implementation. It, however, necessitates co-design facilitators to represent others and anticipate and appropriately facilitate conflicts that stem from participants’ diverse views, interests and goals,^31^ sometimes without possessing the necessary skills.^22^ While several studies have explored participants’ experiences with co-design and its potential benefits, less is known about co-design facilitators’ experiences or their views on its feasibility, usefulness and impact on research practice, policy or patient outcomes.^5 22 32 33^ Existing research tends to focus primarily on the potential benefits of co-design, with limited reporting on its impact on research practice, policy or patient outcomes. Reporting of potential unintended and negative consequences of co-design is particularly lacking.^34 35^

Moreover, there remains a paucity of studies on effective participant engagement strategies—especially involving patients—in healthcare research in low- and middle-income countries (LMICs)^36^,^38^ and marginalised populations,^38^ despite co-design’s potential to address research inequalities.^39^ Public health interventions developed in donor countries without considering local power dynamics, and sociocultural and political realities in LMICs have been criticised for ineffectiveness and perpetuating neoliberal practices.^6^ Thus, co-design is increasingly encouraged in LMICs to address specific population needs in intervention and research design, even in maternity care,^6 40^ though its integration in healthcare research—particularly in maternity care—remains unclear. In 2023, the WHO published an implementation toolkit to support health facilities to systematically implement WHO’s intrapartum and immediate post-natal care recommendations; elements of co-design are a central aspect of this toolkit.^40^ For instance, it emphasises engaging key stakeholders early in implementation and provides practical guidance and recommendations for conducting brainstorming sessions, interviews, focus groups and surveys to help identify barriers and facilitators for change and subsequently identify appropriate implementation strategies. It also promotes interprofessional collaboration and community engagement for long-term sustainability.

While emerging evidence shows growth in co-design use in LMICs, approaches vary, with patients in these settings acting more as advisors than co-participants,^36^ although more recent studies indicate a trend towards more systematic involvement of end users.^6^ Nevertheless, significant challenges remain, including higher patient engagement costs and uncertainty about how to effectively involve patients as co-participants, given co-design’s relative novelty in LMICs.^36^ Given these gaps, we sought to explore co-design facilitators’ perspectives and experiences using co-design to improve intrapartum care in four sub-Saharan African settings over a 2-year period. The inquiry focuses particularly on how they fostered engagement, built trust and addressed unintended consequences during the co-design process.

## Methods

### Study design, setting and study-specific definitions

This qualitative study is part of a multicountry hospital-based quality improvement (QI) co-design project, ALERT (Action Leveraging Evidence to Reduce Perinatal Mortality and Morbidity),^41^ which aims to strengthen intrapartum care through end user participation, midwifery training, leadership mentoring and QI activities. ALERT seeks to promote patient-centred care by giving affected end users an active role in the design and implementation of suitable interventions in close partnership with researchers. The co-design component of ALERT included three phases and numerous work packages (table 1), inspired by the stages of experience-based co-design described by Robert *et al*^20^ and the principles and stages of co-creation described by Leask *et al*.^42^ Study-specific definitions are provided in table 2.

**Table 1:** The three phases in the co-design component of Action Leveraging Evidence to Reduce Perinatal Mortality and Morbidity (ALERT), including associated activities

	Phases	Activities
1	Formative assessment	Interviews with co-design facilitators, maternity care providers (mainly nurses and midwives) and birthing parents. Group discussions with companions of birthing parents. Preliminary data analysis of interviews with co-design facilitators, maternity care providers (mainly nurses and midwives) and birthing parents. Co-design workshops with (a) birthing parents and their companions and (b) maternity care providers only, and led by co-design facilitators to discuss preliminary data and next steps in the intervention design. Maternity care providers were never included in the same co-design workshops as birthing parents and their companions in either of the settings.
2	Stepped-wedge implementation	Feedback was obtained from birthing parents, their companions and maternity care providers during the formative assessment phase about how intrapartum care was being delivered and how it could be improved informed the intrapartum care research priorities. Following these initial activities, maternity care providers compiled lists of necessary quality improvement activities and identified locally available and context-appropriate, low-cost solutions. During the stepped-wedge implementation of these activities, ad hoc co-design workshops, led by co-design facilitators, were convened on a continuous, needs-driven basis. Facilitators organised workshops with relevant end users in selected hospitals to address emerging challenges; for example, when labour companionship of choice was not implemented as intended, a co-design workshop with maternity care providers was held to discuss opportunities and challenges. This iterative approach generated additional quality improvement activities, enabling the intervention and its implementation to evolve in response to real-time feedback and changing needs.
3	Evaluation	Mixed methods process evaluation.

**Table 2:** Study-specific definitions

Terms used	Definitions
Co-design facilitators	Trained research assistants assigned to collect and analyse formative qualitative and quantitative data, as well as facilitate co-design workshops with end users, guiding discussions on various issues identified during formative data collection. This technique was used for end users to propose contextually appropriate solutions. Initial co-design facilitator training was delivered online due to the COVID-19 pandemic using a cascading approach: following general training of all teams, each country team complemented their training with contextualised training sessions. An online platform was used for recorded sessions, cross-country experience sharing and resource exchange. As COVID-19 restrictions eased and the project advanced, co-design facilitators and research leaders continued discussing and reflecting on findings and progress during in-person presentations and writing retreats.
Co-design participants (or end users)	Included the following end users: birthing parents who just delivered at hospital, their companions and maternity care providers (medical doctors, nurses and midwives). All end users were involved in the formative assessment phase, including the initial co-design workshops. Their feedback on how intrapartum care was being delivered and what could be done to improve care during this phase helped determine research priorities for intrapartum care. After these initial activities, maternity care providers developed lists of quality improvement activities they deemed necessary and identified locally available and locally relevant, low-cost solutions. We referred to this as continuous co-design towards end users during the whole project period.
Birthing parents	Persons who delivered their baby at the institutions in which the co-design intervention was being implemented. This term was used to include as inclusive language as possible.

ALERT was launched in 2020 across the maternity units of 16 district-level hospitals, both public and private not-for-profit (faith-based), in Benin, Malawi, Tanzania and Uganda (four per country). Including both public and private not-for-profit hospitals reflects the typical landscape of hospitals in sub-Saharan Africa. At the time, each hospital had at least 2500 births per year, and the involved countries shared many health system characteristics, although distinct differences did exist. For instance, in Benin and Uganda, the predominant midwifery provider is midwives, while in Malawi and Tanzania, it is nurse-midwives. Malawi and Tanzania have strong task-shifting policies in maternity care, resulting in mostly non-physician clinicians performing caesarean sections,^43^ while in Uganda and Benin, caesarean sections are performed exclusively by medical doctors. All maternity care-related services are formally free at point-of-care in governmental facilities in all countries except Benin, although informal payments, due to medicine and equipment shortages, for example, may be incurred by patients. Maternity care-related services at faith-based hospitals are heavily subsidised. Further details on how maternity care is delivered across these settings, including maternal and neonatal outcomes, have been published elsewhere.^41 44^

### Sampling and data collection

Co-design facilitators—trained research assistants supporting data collection and workshops—were first interviewed in 2021 during the formative phase of the co-design process where they reported challenges balancing their multiple roles, including engaging participants, building alliances with hospital management and making the project relevant for birthing parents and their companions. To explore this further, a total population sample of co-design facilitators actively involved in and leading co-design activities in the ALERT project since its launch in 2020 in either of the four sites (n=10) was invited to participate in follow-up interviews via email, with a total of up to two reminders. None were excluded. Participants were informed about the study’s purpose and provided written informed consent prior to participation. Ultimately, all 10 co-design facilitators (9 women and one man) agreed to participate, all of whom had experience working with co-design or qualitative methodologies in similar contexts in their respective countries. They also had good familiarity with the local healthcare context and maternity care. In fact, some worked clinically themselves. Seven had medical/healthcare training before engaging in global health, three had an educational background as social scientists and four had PhDs. Interviewed co-design facilitators worked in Malawi (n=3), Tanzania (n=3), Benin (n=2) and Uganda (n=2). For confidentiality purposes, no further demographics are reported since less than five facilitators were interviewed per country.

Interviews were conducted between December 2022 and January 2023 by an experienced qualitative researcher who had not previously been involved in the ALERT project (EAS-G), using a semistructured interview guide developed by the co-author team, informed by the initial interviews and literature in the area (online supplemental material file 1). The guide covered four areas of interest: (i) the co-design facilitator’s role, (ii) views on co-design and its barriers and facilitators, (iii) creating collaborative and engaging environments in co-design and (iv) ethical aspects of co-design. Interviews were held in English or French on Zoom and lasted 1–1.5 hours. All interviews were audio-recorded with consent and transcribed for analysis. Interviews held in French (n=2) were conducted with a French interpreter and subsequently transcribed and translated by a native French- and English-speaker. These transcripts were subsequently reviewed by the interpreter to ensure no information was lost in translation.

### Data analysis

Data were analysed using reflexive thematic analysis as described by Braun and Clarke.^45^,^47^ After familiarisation with the transcripts and initial noting of ideas, transcripts were coded inductively in Dedoose by EAS-G using semantic coding to identify meaning throughout the dataset. Codes were subsequently collated by EAS-G into candidate themes, and an initial thematic map was generated. During the analysis process, EAS-G and HMA (an anthropologist and experienced qualitative researcher) engaged in continued reflexive dialogue about interpretations, during which candidate themes were refined, defined and named, ultimately generating the final thematic map.

### Patient and public involvement

Patients and their caregivers involved in the co-design workshops were not involved in the current study, although they contributed to the intervention design and implementation of intervention activities within the overall ALERT project.

### Reflexivity statement

To promote transparency in this multicountry collaborative project spanning across high income and LMICs, a reflexivity statement is provided (online supplemental material file 2). It details efforts made to mitigate potential power imbalances and cultural differences across these settings at all stages of the research process.

## Results

**Figure 1:**
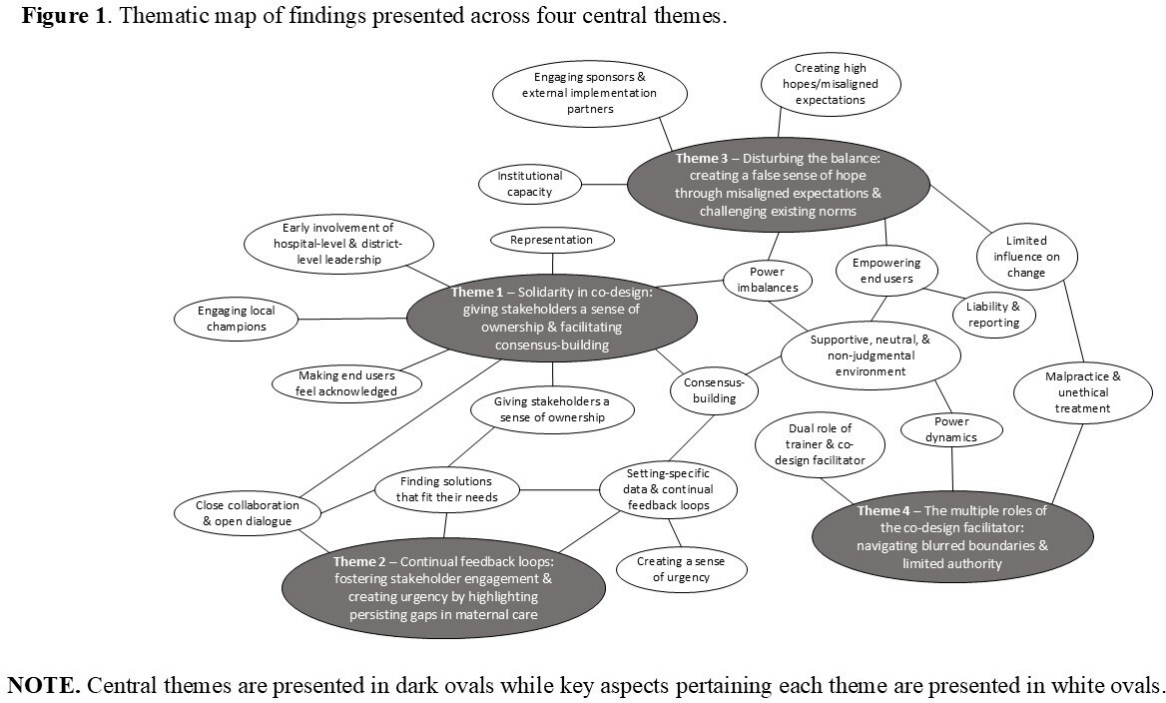
Thematic map of findings presented across four central themes. Note: Central themes are presented in dark ovals while key aspects pertaining to each theme are presented in white ovals.

Facilitators perceived co-design as a highly collaborative process where participants could bring forward contextually relevant solutions to local problems important to them. Nevertheless, this process presented challenges, including appropriately navigating existing norms, power divides, unintended consequences and balancing the numerous roles of the co-design facilitator. Insights are presented across four central themes below and visualised in a thematic map (figure 1).

### Theme 1—solidarity in co-design: giving stakeholders a sense of ownership and facilitating consensus-building

#### Building early partnerships with multiple stakeholder groups

Early project collaboration with hospital and district-level leaders, managers, administrative staff, local champions and end users was considered critical in advancing the co-design process. Leaders were not only given the opportunity to voice their opinions and propose alternative solutions to suggested interventions but also explore and understand factors impacting maternity care beyond the individual health facility.

…we started with management, we just followed the normal procedures, writing a letter, reporting, make a courteous call before anything started and explaining everything […] that we wanted to prepare implementation packages, to support and help improve quality of care, but before that we need to discuss and plan together […] we wanted to design the implementation together […] for healthcare providers to be involved fully. (P8)

Hospital leadership and administrative staff were, however, considered challenging to engage due to competing priorities.

One of the things we’ve tried to do is to meet leaders individually and ensure that they are well-informed about our work in the facility and try to solicit their involvement. (P1)

Early and ongoing engagement across these stakeholder groups was considered important to establish buy-in, foster a sense of ownership and create an environment of mutual trust and respect. The research team and co-design facilitators supported this by building strong, collaborative and non-judgmental relationships with stakeholders from the outset.

Co-design brings that ownership […] end users feel that they have been part of the process and it’s very easy for them to adopt it. I think it can help with sustainability because everything that the end users say is helpful and can be sustained by them… (P1)

#### Facilitating consensus-building while mitigating the power divide

End users were often described as highly engaged and motivated to drive change. Co-design facilitators noted that many providers valued the unique opportunity to voice their opinions in a safe, non-judgemental space, despite existing power imbalances between nurses, midwives and medical doctors that often limited open dialogue. Many co-design facilitators also noted that birthing parents and companions were unaccustomed to being asked to share their concerns, making it harder for some to speak up—especially when interviewed at the hospital—although having companions present helped birthing parents open up.

…they [birthing parents and companions] are difficult to collaborate with because they don't think they have the right to collaborate… […] they are not used to giving their opinion and that is so vital in co-design. (P2)

Aware of this, co-design facilitators emphasised making end users feel heard, recognising them as experts in their own experience and key to finding solutions to improve maternity care. However, providers and birthing parents with their companions were not invited to the same co-design workshops as the power imbalances were perceived too large. These stemmed from the perceived higher social standing of providers, literacy gaps, shared community ties and ongoing care relationships. Co-design facilitators, thus, believed that combining these stakeholder groups would have restricted birthing parents and companions to voice their opinions and caused providers to feel blamed or unappreciated.

…bringing patients to the discussion would have been a challenge […] Most would not be able to speak up in the presence of healthcare workers. Rural people in [country], give respect to their healthcare providers, so not many of them would feel comfortable to voice their concerns. (P7)

Instead, co-design facilitators shared findings from the formative interviews and discussions from the providers’ workshop at the parents and companions’ workshop, and vice versa. This approach allowed participants to express their views, clarify behaviours, reflect on other stakeholders’ experiences and build consensus in a safe space.

The co-design workshops gave room for nurses and clinicians to explain their behaviour. It made nurses understand clinicians, but also clinicians to understand nurses. In the end, I think they were able to come to one common ground. Maybe it also gave them time to reflect and to think about their actions. I don’t know what happened afterwards, but maybe in the end, they were able to understand each other better. (P7)

On reflection, however, several co-design facilitators believed that mixing the groups would have been valuable; a view also shared by some end users.

…since we did these two groups separately, the first group [birthing parents and companions] wanted healthcare providers to be present so that they could be held answerable. When we met healthcare providers, they also felt it could have been nice to combine them with mothers and their companions, so they could also be answerable and maybe explain some things […] however we felt that combining these three groups, there would be a lot of power dynamics. […] But according to them, they wanted to meet face-to-face so that everybody is held accountable for what they had to say. (P4)

### Theme 2—continual feedback loops: fostering stakeholder engagement and creating urgency by highlighting persisting gaps in maternal care

Co-design facilitators maintained close communication and supportive relationships with providers through on-site and virtual mentorship, training and chat groups, thus fostering open dialogue, strong engagement and sustained motivation across sites.

…when I’m travelling to the facilities, we make sure to let them know in advance. They should know our availability so if there is anything that requires us to have a sit-down, people should know so that they can also make themselves available. (P4)

Co-design facilitators also regularly collected, analysed and disseminated data from the electronic birth registry, formative interviews and on-site observations, not only revealing site-specific challenges but also keeping providers engaged.

…when we go for either mentoring or supervision visits, that is when you get different insights, not only from talking to them, but also from observing. […] there are things that you pick up from the environment, from the hospital, from the interactions from women and nurses and things like that. (P6)

Monthly data feedback to facility leaders and providers, but also quarterly visits by co-design facilitators, gave providers, hospital leaders and administrators additional opportunities to discuss challenges and collaboratively identify solutions. To broaden reach, data were presented at co-design workshops with providers and birthing parents with their companions. This was a new experience for end users that helped foster engagement and increased motivation for change.

For the first round of workshops I’d say participants were very engaged. This is something they have never come across before, where people come, collect data, go back, analyse, come back, discuss the findings, and maybe propose possible solutions. […] So, people are really enthusiastic. […] People have a lot to say. It is their chance to express themselves… (P4)

According to the co-design facilitators, the key to the success of this data dissemination was that the data were local and context-specific and, thus, relatable to end users. Data were also provided early in the partnership and in a continual feedback loop, creating a sense of urgency for change. This allowed stakeholders to buy into planned interventions and encouraged them to propose relevant solutions.

### Theme 3—disturbing the balance: creating a false sense of hope through misaligned expectations and challenging existing norms

Some co-design facilitators experienced or reflected on unintended consequences brought by the co-design process, mainly regarding creating a false sense of hope among providers through misaligned expectations and disrupting existing care processes. One central issue raised was the inability (as co-design facilitators) to meet providers’ expectations due to factors beyond their control. Co-design facilitators noted that numerous issues related to limited institutional capacity were observed or raised by providers that could not be addressed by ALERT, such as lack of equipment, structural constraints or large staff turnover.

Institutional capacity is really challenging. I can’t say we have a very good solution. We are really struggling when co-design requires something outside the scope of the intervention, for example, lack of supplies. …we offer knowledge, resources, skills, but what they need is Doppler, money to partition the ward. (P1)

Co-design facilitators perceived the situation challenging to handle, with some expressing feelings of guilt and powerlessness when they were unable to address these identified needs. Co-design facilitators struggled with the tension between wanting to respond to the needs raised by providers, while being constrained by the project’s scope. Only one site reported actively striving to engage local external stakeholders, such as non-governmental organisations, who might be interested in sponsoring the needs arising from institutional constraints that fell outside the research’s scope and funding.

Another concern was that, while they believed it positive that co-design empowers participants to speak up and claim their rights, it can also unintentionally rekindle existing tensions and power imbalances among co-design participants, leading to finger-pointing rather than uniting them to empower each other as victims of the same system. To address this, co-design facilitators reported doing their utmost to create a neutral, safe and trusting environment where co-design participants could share their views without blame, finger-pointing or fear of litigation and to ensure all participants’ concerns were validated during the co-design workshops.

…when we introduced the co-design process to the participants, we said that this is not a blame game. We are not saying nurses are better or clinicians are better. This is just what we found, and we are trying to find a solution. […] that made them all become flexible with speaking about the problem. (P7)

This mitigation by the co-design facilitators was also considered an important aspect of facilitating consensus-building in a context with existing power imbalances among stakeholder groups.

…it was interesting to hear how clinicians and nurses reacted to our pre-implementation findings… […] It created room for them to discuss and reach a level ground for all of them … (P7)

### Theme 4—the multiple roles of the co-design facilitator: navigating blurred boundaries and limited authority

Co-design facilitators often held multiple roles, participating not only in formative work but also on-site QI, staff training and mentorship. Interviewees acknowledged the advantages of being involved in both training and co-design, such as immediately being able to integrate participant insights from the co-design workshops into the training. They also believed that their expertise in the field helped inform the co-design process.

Since we are conducting competency-based trainings, targeting skills and knowledge, it helps that we were part of the co-design, to target those areas which came out as areas of weaknesses both from the healthcare workers and the mothers […] we can incorporate some of the things that came from the co-design meetings into the training. (P1)

However, facilitators recognised that their dual roles and on-site presence could have influenced power dynamics and trust-building between them and end users, thus affecting end users’ willingness to participate, especially providers. Being part of a research team from a well-respected institution with people reputable in the research field and, in some cases, being familiar with local teams after having themselves worked in those settings, was thought to help tremendously in engaging staff in co-design. Nevertheless, some co-design facilitators felt their presence made certain co-design participants uneasy, potentially discouraging them from disclosing sensitive issues for fear of punitive consequences. To address this and to minimise any perceived power imbalances between the co-design facilitators and participants, facilitators actively sought to establish a trusting environment. They emphasised that co-design participants were experts in their own setting and experiences and that their role was to support care improvement, not to penalise. Providers were reassured that discussions were confidential and would not lead to disciplinary action.

…explaining that it is really about getting the problem out, it’s not about punishing […] when you are coming from a training institution, sometimes they feel like you know everything, but just telling them that […] we don’t know their context and their problems, and there’s no right and wrong answer […] it’s all about finding out what can be improved. (P6)

Despite these efforts, co-design facilitators sometimes found themselves caught in the crossfire during site visits, witnessing malpractice or mistreatment of birthing parents and their families, while also observing staff struggling to keep up with their tasks under significant constraints. This created dilemmas about when to intervene and when to step aside as the situation unfolded. Some facilitators with maternal care training acknowledged occasionally stepping in to support staff and birthing parents during labour (eg, when observing a rapidly deteriorating situation, or neglect or violence against a birthing parent), even though it risked jeopardising their positionality as a co-design facilitator.

## Discussion

This study explored co-design facilitators’ experiences using co-design to improve intrapartum care in resource-constrained settings, focusing on how they fostered engagement, built trust and mitigated unintended consequences amid power imbalances. Facilitators described co-design as a collaborative, consensus-driven process that empowers end users and fosters a sense of ownership, despite ongoing challenges with sustaining stakeholder engagement, managing misaligned expectations, navigating tensions and balancing multiple roles while maintaining trust and collaborative environment.

### Mitigating power divides

Co-design is an approach used in implementation research that aims to bring together individuals, groups and organisations as equal partners and co-creators with decision-making power^7^,^9^ to collaboratively develop solutions to shared challenges.^1^,^548^ The belief is that distributing power equitably empowers stakeholders, promotes social mobilisation and improves health outcomes.^48^ However, power dynamics in co-design are shaped by the unique social, structural and cultural contexts^39 48^ and likely varied greatly between end user groups, hospitals and countries involved in ALERT. In such settings, existing power divides may result in superficial co-design participation that fails to fully incorporate stakeholders’ diverse perspectives.^48^ This is particularly true when involvement of disadvantaged groups is tokenistic and does not genuinely influence higher level decision-making.^48^ The complex relationships between researchers and community partners further complicate these dynamics.^48^ For co-design to be effective, meaningful stakeholder engagement and genuine power-sharing between stakeholders and researchers are essential,^3948^,^50^ with co-design facilitators playing a critical role.^2 3^

#### Promoting equal engagement

Mitigating power divides among stakeholders is crucial for creating a safe, respectful and transparent atmosphere during co-design workshops, but also for encouraging equal engagement and fostering a sense of ownership.^1 39 51^ Facilitators in this study strove to balance power by establishing a safe and trusting environment for open dialogue, recognising and valuing each participant’s expertise and using accessible language—all important strategies for mitigating power divides.^39^ Workshop composition and setting also influence power dynamics.^35 48^ While mixed-group workshops can promote equal engagement, in practice, significant power imbalances may hinder open participation. For example, patients may hesitate to voice criticisms of healthcare services in front of providers due to perceived authority gaps, while clinicians may unintentionally dominate discussions, prioritising professional concerns over patient needs.^48^ In this study, workshops were conducted separately with different end user groups as power imbalances were perceived too great, risking discomfort and limited dialogue. Nevertheless, several facilitators recognised the value in mixing stakeholder groups as it can foster rapport and facilitate the exchange of experiences and perspectives. However, efforts to mix stakeholders must be sensitive to existing power dynamics in each context.

Birthing parents in this study were interviewed at the hospital prior to discharge, often while in a vulnerable position and unfamiliar setting where power imbalances are likely heightened. This may have limited the input parents and their companions felt comfortable providing, potentially constraining the ability to adequately address care gaps. Birthing parents may have felt obliged to participate or feared repercussions if they reported malpractices. Ideally, interviews with birthing parents should take place in their own communities or homes, where they feel safer and less influenced by power dynamics.^52^ Involving well-recognised and trusted local community leaders as moderators in the co-design sessions could also help bridge power divides.^39^ Additionally, under-represented communities were less often involved in co-design workshops as they were not specifically sampled during recruitment, which could further marginalise these groups. These communities may also experience more pronounced power dynamics during workshops. The risk of unintended negative consequences for disadvantaged groups is highly context-dependent, however,^48^ underscoring the need for improved guidelines on participatory approaches that prevent further marginalisation^30 39^ and ensure that power dynamics are systematically addressed at all levels of the social ecology.^48^

#### Internal versus external co-design facilitation

In this study, the facilitators’ dual roles as research assistants and trainers with local and technical expertise helped them promptly integrate end user insights into the co-design process and training, and build rapport. While this dual expertise can be advantageous,^2^ familiarity with the context and subject area can be a double-edged sword, and facilitators must remain aware of their positionality. While it is important that facilitators understand context-specific issues, there is a risk of losing impartiality or developing ‘tunnel vision’, potentially influencing the co-design trajectory. External facilitators can offer neutrality and objectivity, creating safer spaces for dialogue and helping balance power dynamics,^2^ but often possess limited contextual knowledge that may hinder their ability to support the development of locally relevant recommendations^53^ and building trust.^54^ Additionally, the financial and logistical burden of external facilitation must be considered, especially in resource-constrained settings.^55^

#### Influence of researchers and the research agenda

Researcher involvement can significantly shape co-design processes and outcomes, influencing end user engagement and issue prioritisation. Facilitators in this study noted that being part of a research team encouraged staff participation but may have also inhibited frank discussions due to fears of punitive consequences. They addressed this by building a trusting environment while affirming co-design participants as experts in their own contexts and emphasising that their role was to support improvement, not to assign blame or impose penalties.

Nonetheless, researchers and/or co-design facilitators may—intentionally or unintentionally—prioritise issues that align with their own research agendas, potentially overlooking end user concerns. Even well-intentioned efforts to empower disadvantaged populations can fall short if differences between lay participants and academic experts are interpreted as misunderstanding, lack of awareness or poor literacy.^56^ Similarly, when key decisions—such as those involving resource allocation or policy—are made in less inclusive settings, the voices of marginalised groups may be overlooked.^48^ Researchers must critically reflect on their own privileges and recognise existing power imbalances among all stakeholder groups. To minimise disparities, all participants should be given equal opportunities to contribute to both the co-design process and the project’s overall governance.^38^

#### Navigating blurred boundaries and limited authority

As noted, co-design facilitators’ familiarity with the local healthcare context and maternity care facilitated co-design activities and helped mitigate power divides. However, when witnessing malpractice or ill-treatment, facilitators often faced uncertainty about their responsibility to intervene. Acting or not acting in such situations can unintentionally reinforce existing power imbalances and erode trust, potentially jeopardising relationships, collaboration and participation in co-design. This ‘grey zone’ highlights the need for clear protocols to safeguard both participants and facilitators while maintaining strong, collaborative relationships among all stakeholders.

### Rapport-building, solidarity and continued engagement

Early involvement and sustained communication throughout the intervention design process were crucial for establishing buy-in among diverse stakeholder groups and fostered meaningful engagement in co-design activities. While the ALERT team intended to maintain ongoing collaboration with hospitals, providers and local authorities, co-design facilitators noted that there were no immediate plans to continue involving birthing parents at this stage. This omission risks positioning parents as unequal partners in the co-design process. Some facilitators argued that, since the intervention primarily targets providers, the input of birthing parents is less critical than that of providers during co-design. However, involving end users—particularly patients and caregivers—beyond the initial design phase is important, particularly during evaluation. Despite its importance, continued engagement with patients after discharge can be challenging due to difficulties in maintaining contact and sustaining participation.^5 11 15 23^ In LMICs, patients are, in fact, often involved as consultants rather than as co-researchers in participatory research activities.^36^ Establishing feedback mechanisms to communicate how participants’ contributions ultimately influenced care can help sustain a sense of empowerment and ownership throughout the entire co-design process.^11^ Ultimately, because providers are there to support birthing parents during delivery, healthcare interventions should be designed not only to be evidence-based but also to meet the needs of the birthing parent.

### Unintended consequences of co-design

While co-design’s benefits are well-documented, few studies report its impact on research, practice, policy or population outcomes.^5 22 23^ As a result, there currently is limited literature exploring the unintended consequences of co-design and a resultant dearth of critical discussion on how to address them.^34 49^ In this study, facilitators identified two important unintended consequences: the risk of unintentionally rekindling existing tensions and power imbalances among stakeholders leading to finger-pointing rather than empowerment, and creating false hope among providers due to misaligned expectations.

#### State and act of ownership

Building early partnerships, fostering trust and mitigating power divides in co-design are instrumental for cultivating a sense of joint ownership among end users—an often-undervalued aspect of participatory research.^42^ While this state of ownership empowers participants—recognising them as equal partners in the research process^42^—translating this ownership into action is often challenging. Many participants leave co-design workshops believing that it is someone else’s responsibility to implement ideas generated. Additionally, system-level constraints, such as limited institutional capacity, can further impede the ability to act on this ownership.^2 39^ This disconnect—possessing a sense of ownership but being unable to act—may lead to frustration or helplessness among both end users and facilitators. In our study, facilitators expressed concern about raising expectations they could not meet, sometimes feeling powerless within the project’s constraints. O’Donoghue *et al*^2^ reported a similar challenge, where facilitators sought to mitigate disappointment by assuring participants that all suggestions raised during co-design workshops were valuable and would be relayed to management, even if not immediately addressed. Demonstrating a genuine commitment to acknowledging all input—even in resource-limited settings—can encourage participants to remain open and innovative when proposing solutions.^2^ In our study, one team attempted to address unmet needs by engaging local external stakeholders to potentially sponsor costs that fell outside the research’s scope and funding. While this approach may offer short-term relief, there remains a pressing need for ongoing efforts to mobilise local leadership and governmental authorities to sustainably bridge these gaps in the long term.

These insights underscore the importance of monitoring for unintended consequences and developing protocols to address them should they arise. As interest in co-design grows, researchers and facilitators should be prepared to inform stakeholders about the potential risks of participation before their involvement. Moreover, given the scarcity of literature on co-design in LMICs,^49^ particularly regarding unintended consequences,^36^ further research is needed to understand and address the potential unintended consequences of co-design to end users in diverse contexts.

### Strengths and limitations

Our study addresses a literature gap by exploring co-design facilitators’ experiences in sub-Saharan Africa working with diverse end user groups in maternal and child health. The study was informed by early facilitator insights, allowing for deeper examination of how facilitators navigate and experience co-design. Notably, due to the COVID-19 pandemic, co-design facilitators interviewed in this study received most training online, potentially influencing workshop delivery; however, their prior co-design experience—bolstered by an online platform for recorded sessions, cross-country discussions, resource sharing and peer engagement during data collection and workshops—helped mitigate this. Data collection and analysis were conducted by a qualitative researcher not previously involved in the project to enhance critical reflection. While this outsider perspective promoted reflexivity, it is important to note that differences in the researcher and participant backgrounds may have influenced data collection and interpretation, although the researcher actively considered their positionality throughout the study. A further limitation is that interviews were conducted via Zoom—often audio only due to connectivity issues—which may have limited rapport-building and restricted observation of non-verbal cues.

## CONCLUSION

Since the launch of ALERT, interest in co-design use in LMICs has increased significantly, even in maternity care, yet its integration in healthcare research remains unclear. Given co-design’s relative novelty in LMICs, its approaches vary across studies and settings, making it critical to understand how it is being employed and the challenges faced in these settings. Co-design facilitators in our study perceived co-design as a highly collaborative approach that enables end users to contribute contextually relevant solutions to locally important challenges. However, the process posed significant challenges, such as navigating social norms, addressing power imbalances, managing consequences and balancing the multiple roles of the facilitator. Our study highlights the importance of power-sharing, fostering a sense of ownership and ensuring equitable and sustained engagement of end users throughout the process. It also underscores the need to remain vigilant about the potential unintended consequences of co-design. Further research is needed to deepen understanding of how facilitators influence the design process, including any unintended consequences on stakeholders during and after co-design activities for intrapartum interventions in LMICs.

## Supplementary material

10.1136/bmjopen-2025-109931 online supplemental file 1

10.1136/bmjopen-2025-109931 online supplemental file 2

## Data Availability

Data are available upon reasonable request.

## References

[1] Kirk J, Bandholm T, Andersen O (2021). Challenges in co-designing an intervention to increase mobility in older patients: a qualitative study. J Health Organ Manag.

[2] O’Donoghue F, O’Donnell MT, Griffin TP (2025). Enablers and Barriers to an Experience-Based Co-Design Process to Develop Service Improvements in Enhanced Community Care in Ireland: A Qualitative Study. Health Expect.

[3] Ramos M, Bowen S, Wright PC (2020). Experience based co-design in healthcare services: an analysis of projects barriers and enablers. *Design for Health*.

[4] Noorbergen TJ, Adam MTP, Teubner T (2021). Using Co-design in Mobile Health System Development: A Qualitative Study With Experts in Co-design and Mobile Health System Development. JMIR Mhealth Uhealth.

[5] Brett J, Staniszewska S, Mockford C (2014). Mapping the impact of patient and public involvement on health and social care research: a systematic review. Health Expect.

[6] Longworth GR, Erikowa-Orighoye O, Anieto EM (2024). Conducting co-creation for public health in low and middle-income countries: a systematic review and key informant perspectives on implementation barriers and facilitators. *Global Health*.

[7] Robert G, Ziebland S, Coulter A, Calabrese JD (2013). Participatory Action Research: Using Experience-Based Co-Design to Improve the Quality of Healthcare Services.

[8] Kaisler RE, Missbach B (2020). Co-creating a patient and public involvement and engagement “how to” guide for researchers. Res Involv Engagem.

[9] Grindell C, Coates E, Croot L (2022). The use of co-production, co-design and co-creation to mobilise knowledge in the management of health conditions: a systematic review. BMC Health Serv Res.

[10] Goodyear-Smith F, Jackson C, Greenhalgh T (2015). Co-design and implementation research: challenges and solutions for ethics committees. BMC Med Ethics.

[11] Warner G, Baghdasaryan Z, Osman F (2021). I felt like a human being’-An exploratory, multi-method study of refugee involvement in the development of mental health intervention research. Health Expect.

[12] Chisholm L, Holttum S, Springham N (2018). Processes in an Experience-Based Co-Design Project With Family Carers in Community Mental Health. Sage Open.

[13] Bernhard G, Mahler C, Seidling HM (2018). Developing a Shared Patient-Centered, Web-Based Medication Platform for Type 2 Diabetes Patients and Their Health Care Providers: Qualitative Study on User Requirements. J Med Internet Res.

[14] Vanderlee E, Aston M, Turner K (2021). Patient-oriented research: A qualitative study of research involvement of parents of children with neurodevelopmental disabilities. J Intellect Disabil.

[15] Bishop AC, Elliott MJ, Cassidy C (2018). Moving patient-oriented research forward: thoughts from the next generation of knowledge translation researchers. *Res Involv Engagem*.

[16] Gagliardi AR, Berta W, Kothari A (2015). Integrated knowledge translation (IKT) in health care: a scoping review. Implementation Sci.

[17] Abookire S, Plover C, Frasso R (2020). Health Design Thinking: An Innovative Approach in Public Health to Defining Problems and Finding Solutions. Front Public Health.

[18] Tittlemier BJ, Cooper J, Steliga D (2022). A scoping review to identify and describe the characteristics of theories, models and frameworks of health research partnerships. Health Res Policy Sys.

[19] Reis RS, Salvo D, Ogilvie D (2016). Scaling up physical activity interventions worldwide: stepping up to larger and smarter approaches to get people moving. The Lancet.

[20] Robert G, Cornwell J, Locock L (2015). Patients and staff as codesigners of healthcare services. BMJ.

[21] Louise L, Annette B (2019). Drawing straight lines along blurred boundaries: qualitative research, patient and public involvement in medical research, co-production and co-design. Evid Policy.

[22] Oliver K, Kothari A, Mays N (2019). The dark side of coproduction: do the costs outweigh the benefits for health research?. Health Res Policy Sys.

[23] Domecq JP, Prutsky G, Elraiyah T (2014). Patient engagement in research: a systematic review. BMC Health Serv Res.

[24] Pallesen KS, Rogers L, Anjara S (2020). A qualitative evaluation of participants’ experiences of using co-design to develop a collective leadership educational intervention for health-care teams. Health Expect.

[25] Curran JA, Cassidy C, Bishop A (2020). Codesigning discharge communication interventions with healthcare providers, youth and parents for emergency practice settings: EDUCATE study protocol. BMJ Open.

[26] Ocloo J, Matthews R (2016). From tokenism to empowerment: progressing patient and public involvement in healthcare improvement. BMJ Qual Saf.

[27] Goodridge D, Isinger T, Rotter T (2018). Patient family advisors’ perspectives on engagement in health-care quality improvement initiatives: Power and partnership. Health Expect.

[28] Castro EM, Malfait S, Van Regenmortel T (2018). Co-design for implementing patient participation in hospital services: A discussion paper. Patient Educ Couns.

[29] Gonzalez M, Phoenix M, Saxena S (2021). Strategies used to engage hard-to-reach populations in childhood disability research: a scoping review. Disabil Rehabil.

[30] Moll S, Wyndham-West M, Mulvale G (2020). Are you really doing “codesign”? Critical reflections when working with vulnerable populations. BMJ Open.

[31] Kazadi K, Lievens A, Mahr D (2016). Stakeholder co-creation during the innovation process: Identifying capabilities for knowledge creation among multiple stakeholders. J Bus Res.

[32] Staley K (2015). “Is it worth doing?” Measuring the impact of patient and public involvement in research. *Res Involv Engagem*.

[33] Gagliardi AR, Kothari A, Graham ID (2017). Research agenda for integrated knowledge translation (IKT) in healthcare: what we know and do not yet know. J Epidemiol Community Health.

[34] Dadich A, Vaughan P, Boydell K (2023). The unintended negative consequences of knowledge translation in healthcare: A systematic scoping review. Health Sociol Rev.

[35] Ní Shé É, Harrison R (2021). Mitigating unintended consequences of co-design in health care. Health Expect.

[36] Ibitoye BM, Garrett B, Ranger M (2023). Conducting Patient-Oriented Research in Low-Income and Middle-Income Countries: A Scoping Review. Patient.

[37] Cook N, Siddiqi N, Twiddy M (2019). Patient and public involvement in health research in low and middle-income countries: a systematic review. BMJ Open.

[38] Vicat-Blanc L, Merry L, Harguindéguy-Lincourt M-C (2025). Co-design of interventions and services with structurally marginalized populations in the context of maternal and early childhood primary care: a rapid scoping review. Prim Health Care Res Dev.

[39] Singh DR, Sah RK, Simkhada B (2023). Potentials and challenges of using co-design in health services research in low- and middle-income countries. *Glob Health Res Policy*.

[40] World Health Organization (2023). Toolkit for Implementation of the WHO Intrapartum Care and Immediate Postnatal Care Recommendations in Health-Care Facilities.

[41] Akuze J, Annerstedt KS, Benova L (2021). Action leveraging evidence to reduce perinatal mortality and morbidity (ALERT): study protocol for a stepped-wedge cluster-randomised trial in Benin, Malawi, Tanzania and Uganda. *BMC Health Serv Res*.

[42] Leask CF, Sandlund M, Skelton DA (2019). Framework, principles and recommendations for utilising participatory methodologies in the co-creation and evaluation of public health interventions. *Res Involv Engagem*.

[43] Mullan F, Frehywot S (2007). Non-physician clinicians in 47 sub-Saharan African countries. Lancet.

[44] El-Halabi S, Annerstedt KS, Agossou C (2025). Labour companionship and respectful treatment of women during childbirth: a cross-sectional study across 16 hospitals in Benin, Malawi, Tanzania and Uganda. *bmjph*.

[45] Braun V, Clarke V (2006). Using thematic analysis in psychology. Qual Res Psychol.

[46] Braun V, Clarke V (2019). Reflecting on reflexive thematic analysis. Qualitative Research in Sport, Exercise and Health.

[47] Braun V, Clarke V, Hayfield N (2019). Handbook of Research Methods in Health Social Sciences. Singapore.

[48] Roura M (2021). The Social Ecology of Power in Participatory Health Research. Qual Health Res.

[49] Redman S, Greenhalgh T, Adedokun L (2021). Co-production of knowledge: the future. BMJ.

[50] Locock L, Robert G, Boaz A (2014). Testing Accelerated Experience-Based Co-Design: A Qualitative Study of Using a National Archive of Patient Experience Narrative Interviews to Promote Rapid Patient-Centred Service Improvement. NIHR Journals Library.

[51] Mburu CW, Wardle CJ, Joolay Y (2018). Co-designing with mothers and neonatal unit staff: use of technology to support mothers of preterm infants.

[52] Reid C, Gee G, Bennetts SK (2022). Using participatory action research to co-design perinatal support strategies for Aboriginal and Torres Strait Islander parents experiencing complex trauma. Women Birth.

[53] Kousgaard MB, Thorsen T (2012). Positive experiences with a specialist as facilitator in general practice. Dan Med J.

[54] Connolly SL, Sullivan JL, Ritchie MJ (2020). External facilitators’ perceptions of internal facilitation skills during implementation of collaborative care for mental health teams: a qualitative analysis informed by the i-PARIHS framework. BMC Health Serv Res.

[55] Smith SN, Liebrecht CM, Bauer MS (2020). Comparative effectiveness of external vs blended facilitation on collaborative care model implementation in slow-implementer community practices. Health Serv Res.

[56] Rawson TM, Castro‐Sánchez E, Charani E (2018). Involving citizens in priority setting for public health research: Implementation in infection research. Health Expect.

