# Supplementary material for: Facilitators’ experiences of co-designing an intrapartum care intervention in four sub-Saharan African countries: a qualitative study
Source: BMJ Open. 2026 Mar 10;16(3):e109931. doi: 10.1136/bmjopen-2025-109931 (PMC12983842; doi:10.1136/bmjopen-2025-109931)
Supplement: online supplemental file 1 [file bmjopen-16-3-s001.docx]

# **SUPPLEMENTARY MATERIAL 1: INTERVIEW GUIDE**

**Interviewee’s role**

1. Tell me about yourself and your role within ALERT.
2. How have you been involved in the co-design process for ALERT?

- How did you contribute to the formative data collection phase?
- How did you contribute to the co-design workshop?
- How is your role as a co-design facilitator similar/dissimilar to your role as a trainer?
- How is your role as a co-design facilitator similar/dissimilar to your role as a QI implementer?

**Perceptions of and experiences with co-design**

1. What does co-design mean to you?
2. What are the main barriers of co-design that you have encountered so far and how have you overcome them?
3. What are the main facilitators of co-design that you have encountered so far? How have they helped the process?

**Engagement and collaboration in co-design**

1. From whom and under what circumstances do you gain most insight from to inform the co-design process in your setting?

- How do you best acquire insights into how things are working/not working?
- Which end-users do you learn from?

1. How do you establish a collaborative atmosphere in the co-design process?
2. From what I understand, some hospitals may not yet be involved in the co-design processes at all while others are. How have you developed and maintained collaborative relationships with hospitals that have not yet been involved in the initial co-design processes versus with those who have been involved?
3. How have you developed and maintained collaborative relationships with participants involved in the co-design process?

- How well have co-design participants engaged in co-design so far?
- Were there times when co-design participants were more/less engaged? Can you please explain?
- What do you think contributes to participants being more/less engaged?
  - How do you encourage/maintain engagement?
  - What are the biggest challenges and facilitating factors for engagement?
  - What suggestions do you have to overcome lack of engagement?
- How do you achieve consensus among end-users?
  - Would you combine mothers, companions, and maternity care providers in one workshop? Why/why not?
- When change ideas are discussed with maternity care providers, how are women and companions referred to?
  - Can you give some examples of when maternity care providers discuss mothers during change idea development?
  - How would maternity care providers and other key stakeholders react if you suggested to involve mothers and companions in this next phase of co-design, i.e., to co-develop the change ideas/interventions?
- How will you involve end-users during the implementation and evaluation phases of ALERT?

**Ethical aspects of co-design**

1. What are some of the ethical challenges you may have come across during your partnership with mothers, companions, and maternity care providers in ALERT? Can you give some examples?

- Have you observed any practices or behaviours that you would consider unethical? What kind of practices or behaviours?

1. How have you addressed or mitigated these ethical challenges?
2. What unintended consequences do you think co-design may bring about?

- Have you experienced any unintended consequences with the co-design process so far? How have you handled them?
- How do you minimize the risk for unintended consequences as part of the co-design process?

1. How do you establish trust and respect with co-design participants?

- What contributes to the lack of trust?
- What can be done to gain more trust?

1. What do you think about power asymmetry/power imbalance in co-design?

- Have you experienced power asymmetry/power imbalance? If yes, how have you dealt with it?

**FINAL REFLECTION, LESSONS LEARNED & WRAP UP**

1. If you had to ask mothers and companions about their experiences with maternity care in the intervention hospitals now, what do you think they would say?
2. Can you think of any point where you had an ‘aha’ moment, or you learned something about co-design that really surprised you?

- What was it? Why was it meaningful or surprising to you?

1. So far, how well have your expectations of using co-design within ALERT been met?
2. How have your perceptions of co-design been impacted since you started implementing this strategy?
3. Before we wrap up, is there something that you would like to add or that you think I should have asked?
