# Supplementary material for: Facilitators’ experiences of co-designing an intrapartum care intervention in four sub-Saharan African countries: a qualitative study
Source: BMJ Open. 2026 Mar 10;16(3):e109931. doi: 10.1136/bmjopen-2025-109931 (PMC12983842; doi:10.1136/bmjopen-2025-109931)
Supplement: online supplemental file 2 [file bmjopen-16-3-s002.docx]

# **SUPPLEMENTARY MATERIAL 2: REFLEXIVITY STATEMENT**

|  | **Question** | **Response** |
| --- | --- | --- |
| **Study conceptualisation** | How does this study address local research and policy priorities? | This study is about the facilitation of codesign processes in four local settings (Benin, Malawi, Tanzania and Uganda) by four local research teams. It attempts to contribute to the understanding of local research practices. |
|  | How were local researchers involved in study design? | One of the co-authors (EC) is based at a University in Malawi; Malawi being one of the four countries included in this study. All authors contributed to the study design. |
| **Research management** | How has funding been used to support the local research team(s)? | The study was funded as part of the ALERT project which is funded by the European Commission’s Horizon 2020 (No 847824). Local research teams in each of the four ALERT countries were partners with their budgets in the project. |
| **Data acquisition and analysis** | How are research staff who conducted data collection acknowledged? | They are acknowledged in the acknowledgements section. |
|  | How have members of the research partnership been provided with access to study data? | Yes, all data is available for the ALERT research team. Data management agreements are established. |
|  | How were data used to develop analytical skills within the partnership? | Analytical skills were developed between partners through regular discussions during the five-year project. EC led capacity building on qualitative analysis skills. |
| **Data interpretation** | How have research partners collaborated in interpreting study data? | The content of this research was reﬁned based on the substantial contributions and reﬂections by all collaborating research partners. It was also discussed at a writing retreat. |
| **Drafting and revising for intellectual content** | How were research partners supported to develop writing skills? | All research partners contributed to the writing of this analysis. During the ALERT project period, junior researchers were supported through online and face-to-face seminars and one face-to-face writing retreat. |
|  | How will research products be shared to address local needs? | The ﬁndings will be disseminated though open access publication at BMJ Global Health in addition to widespread sharing via all partner universities, the ALERT website and social media channels. |
| **Authorship**  **Training** | How is the leadership, contribution and ownership of this work by LMIC researchers recognised within the authorship? | EC is conducting research in an LMIC. |
|  | How have early career researchers across the partnership been included within the authorship team? | One of the co-authors (NSRN) is an early career researcher who wrote her master thesis on data from one of the included countries (Benin). Authorship is ordered based on the contributions to the study and manuscript writing. |
|  | How has gender balance been addressed within the authorship? | All co-authors are female researchers. |
|  | How has the project contributed to training of LMIC researchers? | The study has contributed to the understanding of codesign, an approach and way of thinking about user experiences during the development and implementation of new initiatives, in LMIC. |
| **Infrastructure** | How has the project contributed to improvements in local infrastructure? | The ALERT project has funded local data storage and management, licenses for analysis and technology. |
| **Governance** | What safeguarding procedures were used to protect local study participants and researchers? | The ALERT project has developed ethical guidelines for collecting and managing data in LMICs. An ethical advisory board read and commented on reports on project activities during the five years of the project. |
