# Supplementary material for: Facilitators’ experiences of co-designing an intrapartum care intervention in four sub-Saharan African countries: a qualitative study
Source: BMJ Open. 2026 Mar 10;16(3):e109931. doi: 10.1136/bmjopen-2025-109931 (PMC12983842; doi:10.1136/bmjopen-2025-109931)
Supplement: Supplementary file 3 [file bmjopen-2025-109931.reviewer_comments.pdf]

PEER REVIEW HISTORY

BMJ Open publishes all reviews undertaken for accepted manuscripts. Reviewers are asked to complete a checklist review form and are provided with free text boxes to elaborate on their assessment. These free text comments are reproduced below.

ARTICLE DETAILS

Title (Provisional)

Facilitators' Experiences of Co-designing an Intrapartum Care Intervention in Four Sub-Saharan African Countries: A Qualitative Study

Authors

Saliba-Gustafsson, Erika A; Rodriguez Neufeld, Nicole Sofia; Hanson, Claudia; Chipeta, Effie; Alvesson, Helle Molsted

---

VERSION 1 - REVIEW

**Reviewer**	**1**
**Name**	**Sulaman, Hira**
**Affiliation**	**United Medical and Dental College, OBGYN**
**Date**	**21-Oct-2025**
**COI**	**None**

---

why the intrapartum care is mentioned why it was not done during antenatal care , if there is no complication in baby delivery how birthing parents can involved in co-design , what they were asked for during intrapartum care, how trust and bonding can be develop in this period , after delivery usual stay is not more than 3 days after c-section . number of participants (demographic tables?) , possible to make tables or charts its difficult to go through all the text and understand , Provide more clarity in relation to maternity care

---

**Reviewer**	**2**
**Name**	**Colomar, Mercedes**
**Affiliation**	**Montevideo Clinical and Epidemiological Research Unit**
**Date**	**20-Nov-2025**
**COI**	**None**

Congratulations on this excellent work. It provides meaningful evidence on a very important topic, and I truly enjoyed reading it. I would love to better understand the level of involvement and training of the co-design facilitators. How many facilitators have been

working across the four sites since the beginning of the project, and for how long had they been working in those settings? I understand that you interviewed ten of them, and that these interviews form the basis of the analysis. How were they distributed across sites? And are the sites similar in terms of the populations they serve and the services they offer?

In the section on “navigation boundaries,” you note that “co-design facilitators’ familiarity with the local healthcare context and maternity care facilitated co-design activities and helped mitigate power divides.” This is a valuable point, and I think readers would benefit from having this information included in the Methods section to better understand the context in which the work took place.

You might also consider briefly referring to the Toolkit for implementation of the WHO intrapartum care and immediate postnatal care recommendations in health-care facilities. This guide, published by WHO in 2023, was designed to support facilities in implementing WHO recommendations in a systematic and participatory way. Co-design is an integral part of the toolkit.

The document highlights the importance of involving key stakeholders from the very beginning—implementers, health workers whose practices are expected to change, as well as women, families and community members. It offers practical tools for brainstorming sessions, interviews, focus groups, and surveys, to help identify barriers and facilitators for change. It also encourages interprofessional collaboration and communication to support long-term sustainability. Its participatory approach aims to adapt strategies to local needs and contexts, while offering guidance on how to navigate challenges that may arise along the way.

A short mention of this toolkit in the Discussion section could help connect your findings with existing global implementation guidance.

**Reviewer**	**3**
**Name**	**Rumbold, Alice R.**
**Affiliation**	**South Australia Health, Women and Kids Theme**
**Date**	**12-Dec-2025**
**COI**	**None**

This paper describes a small qualitative study involving 10 participants across 4 countries. Semi structured interviews were conducted to understand the experiences of co-design facilitators and generate insights about how they fostered engagement, built trust and managed unintended consequences. While the paper addresses an important aim, it would benefit from providing further detail about the co-design process and methods/analysis including additional exemplar quotes, as described below:

(1) Was there an underpinning theoretical approach to the co-design process e.g. participatory action research? And if so, how did this inform the co-design process?

(2) Further detail on what end users were actually able to influence throughout the co-design would be helpful in understanding how co-design was approached. For example, were end users involved in determining research priorities, were they provided with a list of suitable interventions or were these derived solely from the workshops? The finding of potential for misaligned expectations in Theme 3 could reflect a lack of engagement of the end users in setting the priorities for the research.

(3) What type of facilitation training did the co-design facilitators receive? It strikes me as possible that Theme 3– related to misaligned expectations could reflect the level of training/experience of the facilitators in describing the scope of the formative assessments. This should also be discussed as a potential limitation.

(4) Purposing sampling is mentioned, which is generally used to obtain diversity within a given population – what characteristics were the interviewers sampling on? Were diverse perspectives represented?

(5) Did both authors undertake coding of all manuscripts? And was this undertaken independently?

(6) Please include some description of the participations to help orient the reader. To protect privacy, this does not need to be broken down by country and could be presented as a range.

(7) Across Theme's 1. 3 and 4 there is a discussion of creating safe environments. None, of the indicative quotes reflect this, so it would be helpful to include some further quotes to support these statements. Further, how did the participants describe how they created safe and accessible spaces beyond 'making participants feel heard'. E.g. did they allow flexible participation, what facilitation techniques were used to reduce power imbalances, did they build in opportunities to check in with participants and adjust approach accordingly etc. Similarly, 'early engagement' is mentioned in Theme 1 – what is meant by this/ how did participants describe this?

(8) Theme 4 – while interesting, I found it difficult to link the concept of multiple roles back to the original research aim of understanding how to foster engagement, build trust and manage unintended consequences. Further explanation of this is warranted. In addition, the description of the theme includes 'limited authority' yet this is further explained or supported by quotes in the subsequent section, which instead focuses on multiple roles influencing the positionality of the facilitator. What is meant here by 'limited authority'?

(9) The discussion includes the statement 'facilitators noted that underrepresented communities were less often involved' – has this finding been published and could therefore be references? This statement seems to introduce a new finding into the Discussion.

(10) Further elaboration of the study limitations, particularly in relation to points 2, 3 and 4 above is warranted.

VERSION 1 - AUTHOR RESPONSE

Reviewer 1s' comments to the authors	Authors' comments
Why the intrapartum care is mentioned why it was not done during antenatal care, if there is no complication in baby delivery how birthing parents can involved in co-design, what they were asked for during intrapartum care, how trust and bonding can be develop in this period, after delivery usual stay is not more than 3 days after c-section.	The ALERT project (Action Leveraging Evidence to Reduce Perinatal Mortality and Morbidity) is a hospital maternity-based quality improvement project conducted in Benin, Malawi, Tanzania and Uganda, which aims to reduce in-facility mortality and morbidity by strengthening intrapartum care through the development, implementation and evaluation of a multifaceted evidence-based intervention shaped through co-design efforts. For this reason, co-design activities in this project took place in maternity wards and are therefore primarily focused on intrapartum care at the hospital's maternity care units, and not antenatal care.<br><br>While rightly so, persons who have delivered a baby typically will not remain in the hospital for a lengthy period of time, they still are key informants who can provide critical insights into how intrapartum care is being delivered at the hospital where they gave birth. We recognize however, and even point out in the manuscript, (lines 420-426) that involving a birthing parent in co-design workshops soon after delivery to provide insights on the care they received during the intrapartum period is not ideal, and it would have been more optimal for these parents to have been involved in workshops within their own communities post-discharge instead.
Number of participants (demographic tables?), possible to make tables or charts it's difficult to go through all the text and understand.	All information regarding the participants and a brief description of any relevant demographics are now presented in the methods section (lines 180-186).
Provide more clarity in relation to maternity care.	Understanding the maternity care context is indeed important and has been described in previous publications. We have added these citations to the methods sections as well as further description of maternity care provision in these settings (lines 155-169)

Reviewer 2s' comments to the authors	Authors' comments
Congratulations on this excellent work. It provides meaningful evidence on a very important topic, and I truly enjoyed reading it. I would love to better understand the level of involvement and training of the co-design facilitators. How many facilitators have been working across the four sites since the beginning of the project, and for how long had they been working in those settings? I understand that you interviewed ten of them,	Thank you for your wonderful feedback. Regarding, the co-design facilitators and their level of training and involvement in the project, we interviewed all co-design facilitators involved in the project for the purpose of this study. Each of the four countries had two to three co-design facilitators each (lines 185-186). The facilitators started working with the project from its launch in 2020, but all facilitators also had

and that these interviews form the basis of the analysis. How were they distributed across sites? And are the sites similar in terms of the populations they serve and the services they offer?	experience working with co-design or qualitative methodologies in similar contexts in their respective countries.<br><br>Facilitator training was conducted during the COVID-19 pandemic therefore, the initial training was conducted online. It followed a cascading approach: after general training of all teams, each country team was complemented with contextualized training sessions. An online platform was used for recorded sessions, cross-country exchanges, and sharing of resources.
In the section on "navigation boundaries," you note that "*co-design facilitators' familiarity with the local healthcare context and maternity care facilitated co-design activities and helped mitigate power divides.*" This is a valuable point, and I think readers would benefit from having this information included in the Methods section to better understand the context in which the work took place.	Thank you for bringing this good point to our attention. We have now added a short description of this in the methods section (in addition to some more demographic details on the interviewees). Specifically, all facilitators "had good familiarity with the local healthcare context and maternity care. In fact, some worked clinically themselves." (lines 180-186).
You might also consider briefly referring to the *Toolkit for implementation of the WHO intrapartum care and immediate postnatal care recommendations in health-care facilities*. This guide, published by WHO in 2023, was designed to support facilities in implementing WHO recommendations in a systematic and participatory way. Co-design is an integral part of the toolkit. The document highlights the importance of involving key stakeholders from the very beginning—implementers, health workers whose practices are expected to change, as well as women, families and community members. It offers practical tools for brainstorming sessions, interviews, focus groups, and surveys, to help identify barriers and facilitators for change. It also encourages interprofessional collaboration and communication to support long-term sustainability. Its participatory approach aims to adapt strategies to local needs and contexts, while offering guidance on how to navigate challenges that may arise along the way. A short mention of this toolkit in the Discussion section could help connect your findings with existing global implementation guidance.	Thank you for pointing out to us to cite this important report. We have now cited it in the introduction (lines 126-133).

**Reviewer 3s' comments to the authors**	**Authors' comments**
This paper describes a small qualitative study involving 10 participants across 4 countries. Semi structured interviews were conducted to understand the experiences of co-design facilitators and generate insights about how	

they fostered engagement, built trust and managed unintended consequences. While the paper addresses an important aim, it would benefit from providing further detail about the co-design process and methods/analysis including additional exemplar quotes, as described below:	
(1) Was there an underpinning theoretical approach to the co-design process e.g. participatory action research? And if so, how did this inform the co-design process?	The steps in experience-based co-design inspired us (Robert G, et al. Patients and staff as codesigners of healthcare services. *BMJ* 2015; 350:g7714) and we were also guided by the understanding of co-design by Leask et al. (Leask CF, et al.) Framework, principles and recommendations for utilising participatory methodologies in the co-creation and evaluation of public health interventions. *Res Involv Engagem*. 2019; 9:2. The initial data collections were important to understand some of the problems that end-users were experiencing. It also informed the decision to maintain a dialogue with reflexive sessions, especially with healthcare professionals (HCPs), throughout the project period.
(2) Further detail on what end users were actually able to influence throughout the co-design would be helpful in understanding how co-design was approached. For example, were end users involved in determining research priorities, were they provided with a list of suitable interventions or were these derived solely from the workshops? The finding of potential for misaligned expectations in Theme 3 could reflect a lack of engagement of the end users in setting the priorities for the research.	The role of the end-users changed during the course of the project. Initially, end-users provided feedback on how intrapartum care was being delivered and what could be done to improve care during these co-design workshops. After these initial activities, HCPs developed lists of quality improvement activities for which they saw a need and identified locally low-cost, suitable solutions. We referred to this as continuous co-design towards end-users during the whole project period.
(3) What type of facilitation training did the co-design facilitators receive? It strikes me as possible that Theme 3– related to misaligned expectations could reflect the level of training/experience of the facilitators in describing the scope of the formative assessments. This should also be discussed as a potential limitation.	Facilitator training was conducted during the COVID-19 pandemic therefore the initial training was conducted online. It followed a cascading approach: after general training of all teams, each country team complemented with contextualized training sessions. An online platform was used for recorded sessions, cross-country exchanges and sharing of resources. All co-design facilitators had been involved in the project since its launch in 2020 but also had previous experience working with co-design and/or other qualitative methods in similar contexts in their respective countries.<br><br>It is unclear in our study whether facilitators were upfront with participants regarding their ability to address certain pressing issues that arose in the workshops, although as mentioned in the discussion

	(lines 511-514), one team did attempt to address unmet needs that arose during the workshops by engaging local external stakeholders to potentially sponsor costs that fell outside the research's scope and funding, which might work in the short-term but likely not in the long-term.
(4) Purposing sampling is mentioned, which is generally used to obtain diversity within a given population – what characteristics were the interviewers sampling on? Were diverse perspectives represented?	We have not rectified the text (lines 180-186) to better describe our sample. All the co-design facilitators working on the project at the different sites (n=10) were interviewed for the purpose of this study.
(5) Did both authors undertake coding of all manuscripts? And was this undertaken independently?	Interviews were coded independently by one co-author (EAS-G). This has now been clarified in the text (lines 199-206). This approach (i.e., analysis conducted by a single qualitative analyst) is consistent with how Braun and Clarke conceptualise reflexive thematic analysis. In their view, the researcher's subjectivity and interpretive engagement with the data is considered a central analytic resource. Reflexive thematic analysis assumes that themes are actively generated by the analyst through deep, iterative engagement with the data, not discovered as objective entities. This makes it well suited to be conducted by one researcher who can document and reflect on their decisions throughout. Having multiple analysts is optional rather than a requirement in reflexive thematic analysis, however since it can help broaden interpretive perspectives, we chose to involve co-authors in collaborative, reflexive dialogue about interpretations rather than striving for coding consensus.
(6) Please include some description of the participations to help orient the reader. To protect privacy, this does not need to be broken down by country and could be presented as a range.	Thank you for your comment. Almost all facilitators were women (n=9), seven had medical/healthcare training before engaging in global health while three had an educational background as social scientists. Four had PhDs. This information has now been included in the methods section, lines 180-186.
(7) Across Theme's 1, 3 and 4 there is a discussion of creating safe environments. None, of the indicative quotes reflect this, so it would be helpful to include some further quotes to support these statements. Further, how did the participants describe how they created safe and accessible spaces beyond 'making participants feel heard'. E.g. did they allow flexible participation, what facilitation techniques were used to reduce power imbalances, did they build in	Thank you for your important reflection. To elucidate how co-design facilitators attempted to create a safe space for open dialogue between nurses and clinicians, when power dynamics were typically high, we presented the following quote in Theme 3: "...when we introduced the co-design process to the participants, we said that this is not a blame game. We are not saying nurses are better or clinicians are better. This is just what we found, and we are trying

opportunities to check in with participants and adjust approach accordingly etc. Similarly, 'early engagement' is mentioned in Theme 1 – what is meant by this/how did participants describe this?	to find a solution. [...] that made them all become flexible with speaking about the problem." (P7) (lines 342-345) It exemplifies how co-design facilitators ensured to make it clear to participants that the purpose of the co-design session is not to point fingers at one another, but rather to find a joint solution that could work for everyone involved. This helped create a safer space for dialogue.<br><br>Another quote in theme 4 also attempts to further showcase how co-design facilitators attempted to help participants feel comfortable to share their views in a safe environment: "We explained that it is really about understanding the problem not about punishing people […] …when you come from a training institution, sometimes people seem to think you know everything, but we tell them we cannot know everything, their context and their problems, and that there's no right or wrong answer […] it's all about finding out what can be improved." (P6) (lines 373-376). Similar to the previous quote, co-design facilitators in this setting, aware that the potential power divide between them as academics and clinical staff could restrict open dialogue, reiterated that their goal was not to punish but rather to give them to opportunity to come with solutions to clinical problems experienced within their own setting, a setting in which they are the expert.<br><br>These were the predominant ways that co-design facilitators expressed they used to create a safe space for open dialogue.<br><br>With regards to *flexible participation*, mothers and caregivers were able to provide their informed consent as to whether they wished to participate in the co-design workshops while for clinicians the approach to involve them in the workshops varied more by site. In some cases, participating in the co-design workshop was a request for more senior leadership which could have led some clinicians to feel that they were required to participate, however we have no data to support this.<br><br>Regarding, *early engagement*, this was described as involving and collaborating with various stakeholders in the project early in the order to build partnerships where stakeholders also had a seat at the table and could propose alternative solutions to

	suggested interventions. We added a quote to reflect this: "…we started with the management, we just followed the normal procedures, writing a letter, reporting, make a courteous call before anything and explaining everything […] that we wanted to prepare implementation packages, to support and help improve quality of care, but that before that we need to discuss and plan together […] we wanted to design the implementation together […] for healthcare providers to be involved fully." (P8) (lines 233-237)
(8) Theme 4 – while interesting, I found it difficult to link the concept of multiple roles back to the original research aim of understanding how to foster engagement, build trust and manage unintended consequences. Further explanation of this is warranted. In addition, the description of the theme includes 'limited authority' yet this is further explained or supported by quotes in the subsequent section, which instead focuses on multiple roles influencing the positionality of the facilitator. What is meant here by 'limited authority'?	While we were particularly interested in understanding how the facilitators fostered engagement, built trust and managed unintended consequences (which was reflected in the types of questions we asked), we nevertheless allowed room in our analysis for other findings to be brought to light.<br><br>This particular theme, while fitting more broadly in understanding their general experiences with co-design facilitation, does shed light on how facilitators believed their multiple roles within the project and how they were perceived by co-design participants impacted participant engagement and trust. This is best described in lines 361-372.
(9) The discussion includes the statement 'facilitators noted that underrepresented communities were less often involved' – has this finding been published and could therefore be references? This statement seems to introduce a new finding into the Discussion.	Thank you for drawing our attention this. The text has now been edited for clarity (lines 427-429).
(10) Further elaboration of the study limitations, particularly in relation to points 2, 3 and 4 above is warranted.	The initial training of facilitators was envisioned as in-person training, which was not possible due to COVID-19 travel restrictions. This limitation was addressed through the use of an online platform of resources and digital methodological discussions while facilitators were in the hospitals collecting data. Mobilisation of postpartum women was difficult during the initial phase of the pandemic, so interviews were conducted at the hospital.

VERSION 2 - REVIEW

Reviewer 1

**Name**	**Sulaman, Hira**
**Affiliation**	**United Medical and Dental College, OBGYN**
**Date**	**29-Jan-2026**
**COI**	

It is not actually related to intrapartum care

**Reviewer**	**3**
**Name**	**Rumbold, Alice R.**
**Affiliation**	**South Australia Health, Women and Kids Theme**
**Date**	**11-Feb-2026**
**COI**	

Thank you for submitting a revised manuscript, there are a couple of instances where the authors clarified their approach but did not update the manuscript accordingly. I believe the manuscript would benefit from adding the following details:

1) In methods, include details of how the facilitators were trained;

2) In methods, state the co-design approaches that informed this work (e.g. References by Robert et al and Leask et al);

3) In methods, include the explanation provided about what is meant by 'continuous co-design'; and

4) In the strengths and limitations section, acknowledge the limitations of online training and how this was mitigated.

VERSION 2 - AUTHOR RESPONSE

Reviewer 1s' comments to the authors	Authors' comments
It is not actually related to intrapartum care.	The ALERT project (Action Leveraging Evidence to Reduce Perinatal Mortality and Morbidity) aims to reduce in-facility perinatal mortality and morbidity across four countries (Benin, Malawi, Tanzania and Uganda), by strengthening intrapartum care specifically. This is being done by developing, implementing and evaluating a multifaceted evidence-based intervention shaped through co-design efforts that involve maternity care providers, birthing parents and their companions, all of whom can provide important insights on how intrapartum care services can be improved. These efforts are facilitated by the co-design facilitators interviewed in this study.

**Reviewer 3s' comments to the authors**	**Authors' comments**
Thank you for submitting a revised manuscript, there are a couple of instances where the authors clarified their approach but did not update the manuscript accordingly. I believe the manuscript would benefit from adding the following details:	Thank you for giving us the opportunity to revise our manuscript again. Please find our changes below, as per your recommendations.
(11) In methods, include details of how the facilitators were trained;	We have now included more details of how the co-design facilitators were trained in Table 2. Specifically, we added the following text: "Initial co-design facilitator training was delivered online due to the COVID-19 pandemic using a cascading approach: following general training of all teams, each country team complemented their training with contextualised training sessions. An online platform was used for recorded sessions, cross-country experience sharing, and resource exchange. As COVID-19 restrictions eased and the project advanced, co-design facilitators and research leaders continued discussing and reflecting on findings and progress during in-person presentations and writing retreats."
(12) In methods, state the co-design approaches that informed this work (e.g. References by Robert et al and Leask et al);	These have now been included in the methods section, sub-section "Study design, setting and study-specific definitions".
(13) In methods, include the explanation provided about what is meant by 'continuous co-design'; and	We have now added further detail in Table 1 regarding how the "continuous co-design" process was carried out. Specifically, we included the following text: "Feedback from birthing parents, their companions, and maternity care providers during the exploratory assessment phase about how intrapartum care was being delivered and how it could be improved informed the intrapartum care research priorities. Following these initial activities, maternity care providers compiled lists of necessary quality improvement activities and identified locally-available and context-appropriate, low-cost solutions. During the stepped-wedge implementation of these activities, ad hoc co-design workshops, led by co-design facilitators, were convened on a continuous, needs-driven basis. Facilitators organised workshops with relevant end-users in selected hospitals to address emerging challenges; for example, when labour companionship of choice was not implemented as intended, a co-design workshop with maternity care providers was held to discuss opportunities and challenges. This iterative approach generated additional quality improvement activities, enabling the intervention and its implementation to evolve in response to real-time feedback and changing needs."
(14) In the strengths and limitations section, acknowledge the limitations	A brief description of the limitations of online training and how it was mitigated has now been included in the Strengths and limitations section.

of online training and how this was mitigated.	
